# Impaired Vesical Function

**Published:** 1884-10

**Authors:** Henry Thompson


### Impaired Vesical Function.

The healthy functions of the urinary bladder are, first, its ability to retain a considerable quantity of urine without occasioning any inconvenience to the individual, and, second, its ability to empty itself easily and completely at will. These functions imply elasticity of the coats of the bladder, which is a mechanical attribute, and contractility, which is to be regarded as a "vital" attribute. Various morbid conditions have the effect of impairing these two vesical functions, so that the bladder may be unable to expand and retain a quantity of urine, or it may be unable to contract and expel the whole of it. The chief modes by which this impairment of vesical-retaining function takes place can be divided into three classes, as follows:

Class 1. Enlargement of growth from the prostate, obstructing, more or less completely, the urethra at its entrance to the bladder, and so presenting an obstacle which the expulsive power of the bladder and associated muscles cannot surmount.

Class 2. Loss of nerve-power to the muscles of the organ, constituting paralysis.

Class 3. "Atony" of the structures of the bladder, invalidating their action, a term of doubtful import, and ordinarily so employed as to comprehend all other forms of vesical incompetence not designated by Classes 1 and 2. The first class, constituting prostatic hypertrophy and outgrowth, is familiar to all surgeons and need not be dwelt on. There is one feature of this condition, however, that is sometimes overlooked. The most complete form of organic occlusion at the neck of the bladder may be present, giving rise to the most distressing symptoms, and yet the finger in the rectum is unable to detect any prostatic enlargement. A small prominence at the neck of the bladder is more dangerous than a large hypertrophy; the former often renders the patient absolutely incompetent to empty the bladder, while the latter, though more or less troublesome, seldom causes so much disturbance. In regard to the operation of section or resection of the obstruction, Sir Henry concludes that where the retention has existed for some time, it is highly improbable that it will do any good, and may be even considered dangerous. When the little outgrowth can be dealt with in the early stage of its existence, a division of the tissues *may* overcome the obstruction and restore, wholly or partially, the function of the bladder. But even then the catheter can seldom be dispensed with.

In the second class of cases the impairment of function manifestly depends upon an injured or diseased nerve-center and the treatment must be directed to its restoration before improvement can be expected.

In regard to the third class, Sir Henry mentions three conditions that may give rise to impaired vesical function:

1. Chronic inflammation affecting the tissue beneath or outside the mucous membrane of the bladder. As an example of this, repeated attacks of ordinary cystitis can be taken. A single attack may pass off, leaving the bladder but slightly, if at all, impaired in function, but repeated attacks, by spreading to the sub-mucous and muscular tissues, cause an hypertrophy ot the walls of the bladder which is more or less permanent.

2. Chronic inflammation of the prostate and neck of the bladder usually following gonorrhœa. Repeated attacks of gonorrhœa or want of care in its treatment, or the injudicious use of instruments, may give rise to an inflammation of the prostate with more or less persistent induration and swelling of the organ, causing an impairment of the vesical function of the bladder. While the nature of this enlargement is unrelated to that which constitutes hypertrophy, its effects may be the same. A small amount of urine is retained and troublesome symptoms developed in consequence.

3. The presence of a calculus is a frequent cause of the failure of the bladder to empty itself, and this may persist for some time after the calculus has been successfully removed. This condition is one that is well understood, and the indication for treatment is plain, so that it need not detain us. In regard to the treatment, in general, we quote Professor Thompson's conclusions, viz.: "It follows, from all the preceding considerations, that our chief reliance for successful treatment in all the forms of impaired vesical function which give rise to retained urine, is the habitual use of the catheter; and I must add that the remedy is not only most valuable, but that it is, for the most part, indispensible, and the sooner it is employed in the great majority of cases, the greater is the chance of ultimate recovery, in addition to the immediate relief which the instrument affords.

It is the failure to perceive this fact, especially in relation with the less obvious forms of incompetence, which has led, as I said at the outset, to my choice of the subject. The failure is due not only to the oversight of the pathological condition which demands mechanical aid, but to the strong and very natural disinclination which so widely exists to the employment of instruments—I had almost added to the prejudice which is entertained against them—and I fear that this, also, must be admitted."—*Sir Henry Thompson, F. R. C. S., in British Medical Journal, July* 5, 1884.

